# Prognostic Impact of Pregnancy‐Associated Plasma Protein‐A (PAPP‐A) for Gestational Diabetes Mellitus (GDM): An Updated Systematic Review and Meta‐Analysis of More Than 90 000 Pregnancies

**DOI:** 10.1111/aji.70087

**Published:** 2025-06-06

**Authors:** Ing‐Luen Shyu, Yung‐Chieh Tsai, Tian‐Ni Kuo, Yow‐Ling Shiue

**Affiliations:** ^1^ Department of Obstetrics and Gynecology Chi Mei Medical Center Tainan Taiwan; ^2^ Institute of Biomedical Sciences, College of Medicine National Sun Yat‐sen University Kaohsiung Taiwan; ^3^ Department of Pharmacy Chia Nan University of Pharmacy and Science Tainan Taiwan; ^4^ Institute of Precision Medicine, College of Medicine National Sun Yat‐sen University Kaohsiung Taiwan

**Keywords:** gestational diabetes mellitus (GDM), prediction, pregnancy‐associated plasma protein‐A (PAPP‐A), prognosis

## Abstract

**Objective:**

Gestational diabetes mellitus (GDM) constitutes a significant health concern during pregnancy, warranting a thorough investigation into potential prognostic markers. Pregnancy‐associated plasma protein‐A (PAPP‐A), which is present at high levels during pregnancy, exhibits altered concentrations even before the clinical diagnosis of GDM, highlighting its potential as an early biomarker for this condition. This systematic review and meta‐analysis aimed to review and synthesize the latest evidence comprehensively to explore the correlation between maternal PAPP‐A levels and the development of GDM, drawing from the most recent publications available.

**Methods:**

PubMed, EMBASE, and Cochrane CENTRAL were searched systematically up to June 2, 2024. Cohort and case‐control studies reporting PAPP‐A levels in GDM and non‐GDM women with singleton pregnancy were eligible for inclusion. The quality of studies was measured using the Newcastle‐Ottawa scale (NOS). Standardized mean differences (SMDs) of PAPP‐A levels between GDM and non‐GDM, and odds ratios (ORs) of the association between PAPP‐A and GDM were pooled, with heterogeneity assessed using the Cochran Q test and *I* ^2^ statistic. Sensitivity analysis was employed (PROSPERO ID: CRD42024580169).

**Results:**

Nineteen studies involving 92 200 pregnant women were included in the systematic review and meta‐analysis. The gestational age at sampling varied from 10 to 14 weeks. Meta‐analysis revealed a significantly lower PAPP‐A level among women with GDM compared with those without (pooled SMD = −0.31, 95% CI: −0.56 to −0.06). Furthermore, meta‐analysis revealed that women with a low PAPP‐A level had a significantly higher risk of developing GDM (pooled OR = 1.75, 95% CI: 1.45–2.11). Despite observed publication bias, sensitivity analysis affirmed the robustness of the results.

**Conclusions:**

This updated systematic review and meta‐analysis underscored the prognostic significance of maternal PAPP‐A levels with respect to the development of GDM. Low PAPP‐A level is associated with an increased risk of GDM. These findings advocate for the inclusion of PAPP‐A assessment in the clinical evaluation of GDM risk.

## 1 Introduction

Gestational diabetes mellitus (GDM) is a significant health concern, affecting 15%–25% of pregnant women globally [1]. GDM is the most prevalent metabolic disorder during pregnancy, characterized by glucose intolerance first recognized during gestation, as defined by the American Diabetes Association (ADA) [2]. Further, GDM is associated with both immediate and long‐term maternal and fetal complications. Pregnant women with GDM have an increased risk of developing hypertensive disorders, including gestational hypertension and preeclampsia. Additionally, GDM is linked to adverse perinatal outcomes, such as fetal macrosomia, neonatal hypoglycemia, respiratory distress syndrome, and a higher likelihood of cesarean delivery [3, 4]. Besides, the global prevalence of GDM has been rising, potentially due to lifestyle changes, increasing obesity rates, and increased maternal age [5, 6].

GDM diagnosis relies on oral glucose tolerance tests (OGTT) performed at 24–28 weeks of gestation, which limits early intervention. The Consensus Panel from the International Association of Diabetes in Pregnancy Study Groups (IADPSG) evaluated OGTT cut‐offs based on odds ratios (ORs) of 1.5, 1.75, and 2.0 for fetal macrosomia, neonatal adiposity, and fetal hyperinsulinemia (≥90th percentile). After deliberation, an OR of 1.75 was deemed appropriate from HAPO follow‐up Study (HAPOFUS) [7, 8], corresponding to glucose thresholds of ≥92 mg/dL (5.1 mmol/L) fasting, ≥180 mg/dL (10.0 mmol/L) at 1 h, and ≥153 mg/dL (8.5 mmol/L) at 2 h on a 75 g OGTT [9]. With increasingly heavy health care burden, GDM prevalence has increased by 2–3 times compared to older criteria [9].

Identifying first‐trimester biomarkers associated with GDM risk could enable earlier prediction and management strategies. An area of growing interest in GDM is the investigation of pregnancy‐associated plasma protein‐A (PAPP‐A), measured in the first trimester and as a potential biomarker for early detection and risk stratification [10]. PAPP‐A is a glycoprotein primarily synthesized by the placenta during pregnancy, playing a pivotal role in fetal development through its regulation of insulin‐like growth factors (IGFs), which influence glucose metabolism [11]. The intricate dynamic interplay between PAPP‐A and IGFs suggests a potential mechanistic link between dysregulated PAPP‐A levels and the pathophysiology of GDM [12, 13]. First‐trimester PAPP‐A screening is already performed for Down syndrome [14], making it a cost‐effective option for GDM risk stratification.

Although previous meta‐analyses conducted in 2018 provided valuable insights into the association between PAPP‐A and GDM, the rapidly evolving landscape of clinical research necessitates an updated and comprehensive synthesis of evidence [15, 16]. A deeper understanding of the role of PAPP‐A in the pathophysiology of GDM is crucial for enhancing predictive capabilities, refining prevention strategies, and optimizing disease management. This updated systematic review and meta‐analysis aims to explore the prognostic significance of PAPP‐A in GDM by synthesizing the latest evidence to elucidate the potential utility of PAPP‐A as a predictive biomarker for GDM in clinical practice.

## 2 Methods

### 2.1 Search Strategy

The current updated systematic review and meta‐analysis adhered to the Preferred Reporting Items for Systematic Reviews and Meta‐Analyses (PRISMA) guidelines [17]. We conducted a comprehensive literature search across prominent public databases with PubMed, EMBASE, and Cochrane CENTRAL, employing specific keywords “Pregnancy‐Associated Plasma Protein‐A” and “gestational diabetes mellitus” combined with Boolean operators and by using medical subject‐headings (MeSH) terms where appropriate for studies published up to June 2, 2024. The search string used for the abovementioned databases was as follows:

*(Pregnancy‐Associated Plasma Protein‐A OR PAPP‐A) AND (gestational diabetes mellitus OR GDM)*

Additionally, we conducted a manual search of the reference lists in the included studies to identify any potentially relevant research.

This updated systematic review and meta‐analysis have been registered in PROSPERO (ID: CRD42024580169) to ensure transparency and adherence to systematic review protocols.

### 2.2 Selection Criteria

This updated review was conducted following the PICOS principle, encompassing participants (P), exposure (E), comparisons (C), outcomes (O), and study design (S). Eligible participants were women with a singleton pregnancy (P). The studies investigated the relationship between PAPP‐A levels in women with GDM (case) and those without GDM (control group) (E&C). The primary outcome (O) was the comparison of PAPP‐A levels between the GDM and non‐GDM groups. The eligible studies’ design could be prospective, retrospective cohort studies, or case‐control studies (S).

We excluded review articles, letters, commentaries, editorials, proceeding research, meeting abstracts, case reports, personal communications, and non‐English studies. Eligibility of each study was confirmed by two independent reviewers (I.L. and Y.C.), with a third reviewer (T.N.) consulted for uncertain cases.

### 2.3 Main Outcome Measures and Data Extraction

Primary outcome of interest is the difference in multiples of the median (MOM) PAPP‐A levels between the GDM group and the non‐GDM (control) group. Secondary outcome was an association between MOM PAPP‐A levels and GDM.

From the eligible studies, we (I.L. and Y.C.) extracted the following information: the first author's name, publication year, study design, country of the study, the number of women, mean maternal age, gestational age at sampling, maternal body mass index (BMI), parity, family history of diabetes, and previous history of GDM.

### 2.4 Quality Assessment

We evaluated the quality of the included studies using the Newcastle‐Ottawa scale (NOS), following the recommendations of the Cochrane Non‐Randomized Studies Methods Working Group [18]. The NOS assigns a maximum of nine points to each study, with scoring criteria adapted for cohort and case‐control studies, respectively. For cohort studies, four points are assigned for appropriate selection of cohort participants, two points for comparability of participants in terms of design and analysis, and three points for adequate outcome ascertainment. For case‐control studies, four points are allocated for selection (including adequacy of case definition, representativeness of cases, selection of controls, and definition of controls), two points for comparability, and three points for exposure assessment (including ascertainment of exposure, use of the same method for case and control groups, and non‐response rate). Based on their cumulative score in the NOS scale, we characterized studies as “low risk of bias” if they attained 7 points or more, “some concerns” if they received 4–6 points, and “high risk of bias” if they garnered fewer than 4 points. Two independent reviewers (I.L. and Y.C.) conducted the quality assessment, and a third reviewer (Y.L.) was consulted to resolve any uncertainties.

### 2.5 Statistical Analysis

In this updated systematic review and meta‐analysis, the primary outcome was the difference in MOM PAPP‐A levels between GDM and non‐GDM, and the secondary outcome was the association between MOM PAPP‐A and GDM. The former used standardized mean difference with 95% confidence intervals (CIs), and the latter used OR and 95% CIs. Heterogeneity among the studies was evaluated using the Cochran Q test and *I* ^2^ statistic. Heterogeneity is defined as follows: *I* ^2^ ≤ 25%, low heterogeneity; 25% < *I* ^2^ < 50%, moderate heterogeneity; 50% < *I* ^2^ < 75%, substantial heterogeneity; and *I* ^2^ ≥ 75%, high heterogeneity. If *I* ^2^ was greater than 50%, indicated heterogeneity, and random effects models were used, and when *I* ^2^ was less than 50%, fixed effect models were used [19, 20]. We used a two‐sided test with a significance level of *α* = 0.05 for statistical analysis. Publication bias was evaluated by using funnel plots [21]. A sensitivity analysis was conducted using the leave‐one‐out approach. All analyses were conducted using R Studio 4.3.2 with the packages “meta,” “dmetar,” and “metafor.”

## 3 Results

### 3.1 Study Selection

**FIGURE 1:**
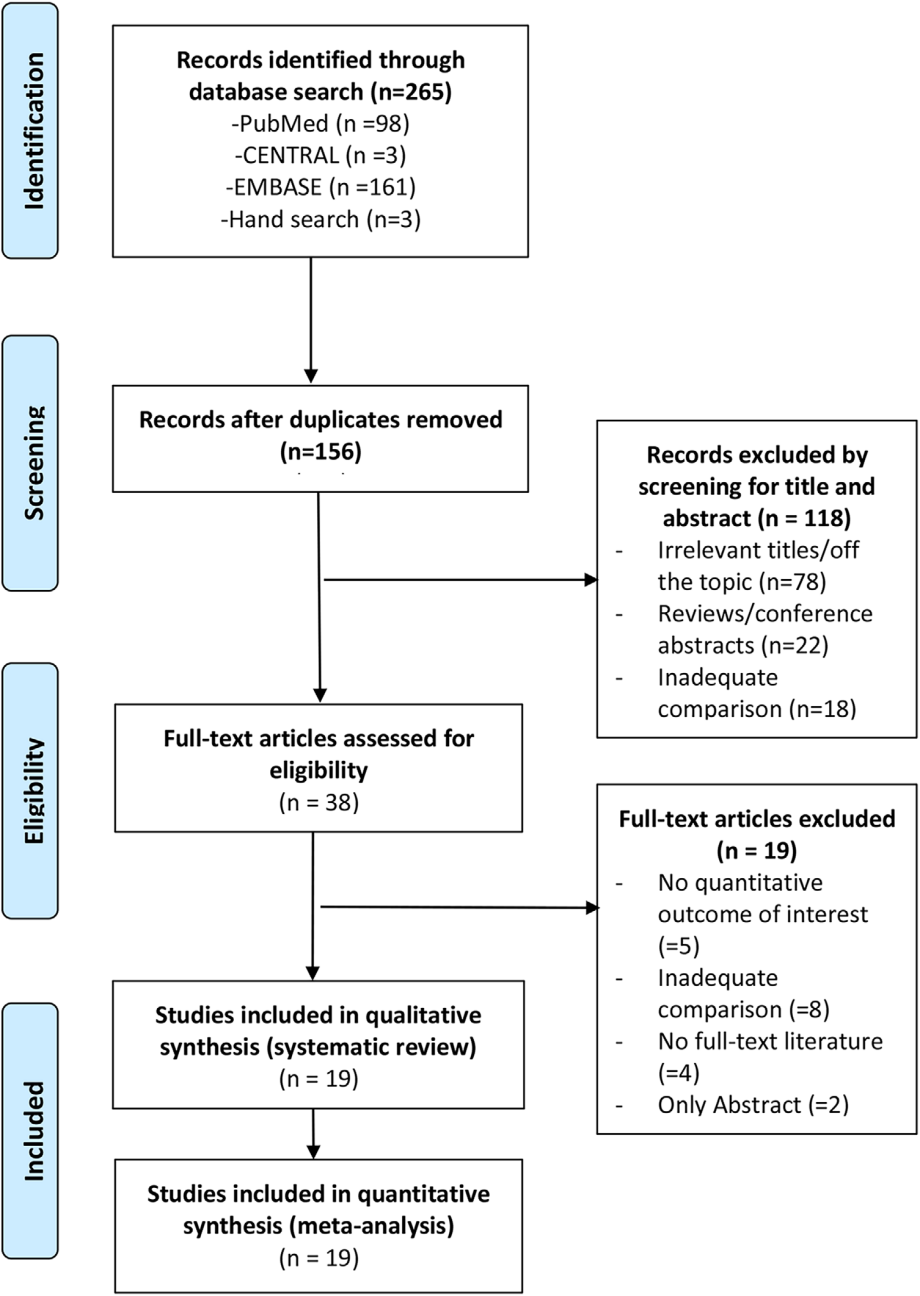
Preferred reporting items for systematic reviews and meta‐analyses (PRISMA) flow diagram of study selection. The numbers of search hits corresponding to each step of the systematic literature search, qualitative review, and quantitative analysis are shown. The reasons for search hit exclusion are described.

A total of 38 full‐text articles were assessed for eligibility, and 19 were excluded. Therefore, 19 studies [10, 22–38] were included in the systematic review and meta‐analysis (Figure 1). A total of 92 200 women with singleton pregnancy were included in meta‐analysis.

### 3.2 Characteristics of Included Studies

The study characteristics are summarized in Table 1. Among these studies, 12 studies [23–28, 30, 33, 35, 37–39] are of case‐control design, 2 studies [29, 34] are prospective studies, and 5 studies [10, 22, 31, 32, 36] are retrospective studies. Nine studies were conducted in Asia, six in Europe, three in Australia, and one in the USA. The mean maternal age ranged from 29.7 to 33.8, and BMI was reported in select studies, with values generally falling between 21.7 and 27.0 kg/m^2^. The gestational age at sampling varied from 10 to 14 weeks, with most studies collecting samples between the 11^th^ and 13^th^ week of gestation. Several studies documented key maternal characteristics, such as nulliparity rates (ranging from 34.2% to 71.1%) and the presence of a family history of diabetes (ranging from 5.4% to 57.1%), while fewer studies reported a previous history of GDM (ranging from 0.5% to 9.0%).

**TABLE 1:** Summary of detailed characteristics of the included studies.

Study	Study design	Country	Total number of women	Number of women (*n*)	Mean Maternal age	Gestational age at sampling, weeks (mean)	Mean BMI, kg/m^2^	Nulliparous (%)	Family history of diabetes (%)	Previous history of GDM (%)	NOS
Cui et al. [24]	Case‐control	China	4872	GDM:750 Non‐GDM:4122	30.5	11–13^th^ week	NA	54.5	5.4	0.5	5
Yildiz et al. [38]	Case‐control	Turkey	378	GDM:207 Non‐GDM:171	30.7	11–13^th^ week	NA	NA	NA	NA	5
Yang et al. [10]	Retrospective	China	518	GDM:318 Non‐GDM:200	30.2	11–13^th^ week	NA	NA	NA	NA	6
Yanachkova et al. [39]	Case‐control	Bulgaria	662	GDM:412 Non‐GDM:250	33.1	10–13^th^ week	24.9	70.1	41.5	NA	5
Tenenbaum‐Gavish et al. [35]	Case‐control	Israel	205	GDM:20 Non‐GDM:185	31.2	GDM:12.7 Non‐GDM:12.6	NA	NA	NA	NA	5
Ramezani et al. [29]	Prospective	Iran	284	GDM:83 Non‐GDM:201	Range:18–35	11–14^th^ week	25.6	NA	NA	NA	6
Ren et al. [30]	Case‐control	China	99	GDM:51 Non‐GDM:48	28.4	GDM:9.87 Non‐GDM:9.93	23.4	49.5	NA	NA	5
Rojas‐Rodriguez et al. [31]	Retrospective	USA	6063	GDM:360 Non‐GDM:5703	29.7	GDM:12.5 Non‐GDM:12.5	NA	43.8	NA	NA	7
Sweeting et al. [33]	Case‐control	Australia	980	GDM:248 Non‐GDM:732	32.3	11–13^th^ week	23.6	56.6	19.3	6.9	6
Xiao et al. [37]	Case‐control	China	1585	GDM:599 Non‐GDM:986	30.1	GDM:12.7 Non‐GDM:12.7	NA	71.1	7.6	NA	5
Farina et al. [25]	Case‐control	Italy	72	GDM:12 Non‐GDM:60	32.3	GDM:12.3 Non‐GDM:12.3	22.8	58.3	NA	NA	6
Cheuk et al. [23]	Case‐control	China	520	GDM:169 Non‐GDM:351	32.7	GDM:12.3 Non‐GDM:12.4	21.7	NA	23.7	9.0	5
Syngelaki et al. [34]	Prospective	UK	31 225	GDM:787 Non‐GDM:30 438	30.4	GDM:12.7 Non‐GDM:12.7	NA	49.3	25.0	NA	7
Wells et al. [36]	Retrospective	Australia	1646	GDM:364 Non‐GDM:1282	33.8	10–14^th^ week	23.2	34.2	NA	4.6	7
Beneventi et al. [22]	Retrospective	Italy	224	GDM:112 Non‐GDM:112	33.2	11–14^th^ week	23.2	50.9	57.1	NA	7
Lovati et al. [28]	Case‐control	Italy	673	GDM:307 Non‐GDM:366	32.9	11–14^th^ week	23.6	NA	53.5	NA	6
Kulaksizoglu et al. [27]	Case‐control	Turkey	120	GDM:60 Non‐GDM:60	32.1	11–14^th^ week	NA	NA	NA	NA	6
Husslein et al. [26]	Case‐control	Australia	288	GDM:72 Non‐GDM:216	32.8	11–14^th^ week	27.00	NA	NA	NA	6
Savvidou et al. [32]	Retrospective	UK	41 786	GDM:779 Non‐GDM:41 007	32.1	12.7	24.28	48.5	NA	NA	7

Abbreviations: BMI, body mass index; GDM, gestational diabetes mellitus; NA, not applicable; NOS, Newcastle‐Ottawa Scale.

### 3.3 Meta‐Analysis

**FIGURE 2:**
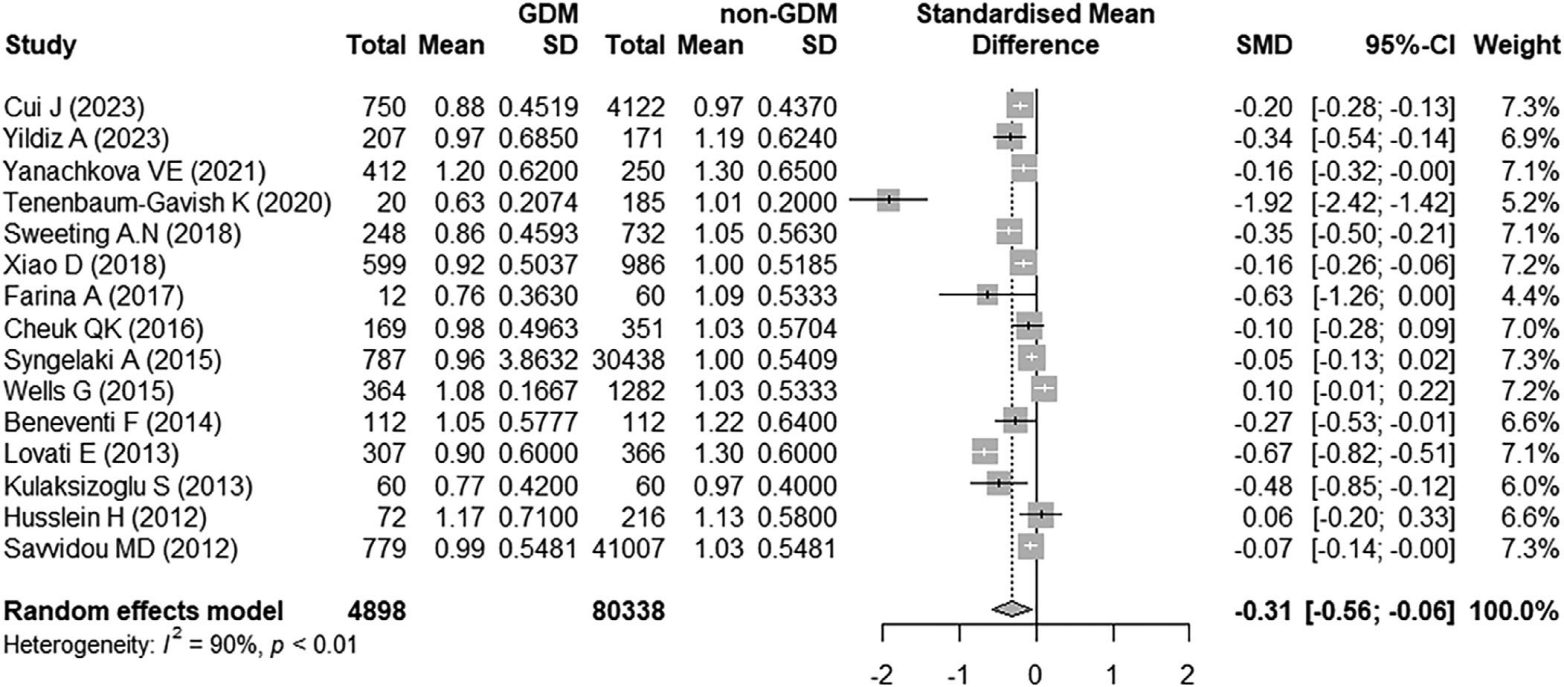
Differences of MOM PAPP‐A levels between gestational diabetes mellitus (GDM) and non‐GDM. CI indicates confidence interval.

Results on the difference of MOM PAPP‐A levels between GDM and non‐GDM are summarized in Figure 2. There were 15 studies [22–28, 32–39] included in the meta‐analysis. High heterogeneity across the studies was observed for this outcome (*I* ^2^ = 90%), and therefore random effects model was used. The analysis weight across these studies ranged from 4.4% to 7.3%. Compared to non‐GDM pregnant women, women with GDM had significantly lower MOM PAPP‐A levels (pooled SMD = −0.31, 95% CI: −0.56 to −0.06).

Pooled association between MOM PAPP‐A and the development of GDM is documented in Figure 4. There were 10 studies [10, 24, 28–31, 33, 36–38] included in the meta‐analysis. The analysis weight across these studies ranged from 0.8% to 18.7%. Besides, women with a low MOM PAPP‐A level had a significantly greater risk of developing GDM (pooled OR = 1.75, 95% CI: 1.45–2.11), with a high heterogeneity detected (*I* ^2^ = 60%) and a random effects model utilized.

### 3.4 Sensitivity Analyses

The sensitivity analysis, conducted through a leave‐one‐out approach, is summarized in Tables 2 and 3. Following the exclusion of each individual study, the pooled results remained consistent in their significance and magnitude, suggesting the pooled results were not largely influenced by any individual study thus are robust.

**TABLE 2:** Sensitivity analyses for differences of MOM PAPP‐A between GDM and non‐GDM women.

Studies left out	SMD (95% CI)	*p* value	tau [2]	tau	*I* ^2^
Cui et al. [24]	−0.32 (−0.59, −0.05)	0.023	0.173	0.416	90.7%
Yildiz et al. [38]	−0.31 (−0.58, −0.04)	0.027	0.173	0.416	90.7%
Yanachkova et al. [39]	−0.33 (−0.6, −0.06)	0.022	0.172	0.414	90.9%
Tenenbaum‐Gavish et al. [35]	−0.21 (−0.33, −0.08)	0.004	0.037	0.193	86.1%
Sweeting et al. [33]	−0.31 (−0.58, −0.04)	0.028	0.173	0.416	90.3%
Xiao et al. [37]	−0.33 (−0.6, −0.06)	0.022	0.172	0.415	90.9%
Farina et al. [25]	−0.3 (−0.56, −0.04)	0.029	0.160	0.400	90.7%
Cheuk et al. [23]	−0.33 (−0.6, −0.06)	0.020	0.169	0.411	90.9%
Syngelaki et al. [34]	−0.33 (−0.6, −0.07)	0.018	0.167	0.408	90.3%
Wells et al. [36]	−0.34 (−0.6, −0.08)	0.013	0.153	0.392	89.4%
Beneventi et al. [22]	−0.32 (−0.59, −0.05)	0.025	0.172	0.415	90.8%
Lovati et al. [28]	−0.28 (−0.55, −0.02)	0.036	0.154	0.393	86.8%
Kulaksizoglu et al. [27]	−0.3 (−0.57, −0.03)	0.030	0.167	0.409	90.7%
Husslein et al. [26]	−0.34 (−0.6, −0.08)	0.015	0.159	0.399	90.7%
Savvidou et al. [32]	−0.33 (−0.6, −0.06)	0.019	0.168	0.410	90.5%

Abbreviations: CI, confidence interval; GDM, gestational diabetes mellitus; PAPP‐A, pregnancy‐associated plasma protein‐A; SMD, standardized mean difference.

**TABLE 3:** Sensitivity analyses for the association between MOM PAPP‐A and GDM.

Studies left out	OR (95% CI)	*p* value	tau2	tau	*I* ^2^
Cui et al. [24]	1.87 (1.51, 2.32)	<0.001	0.045	0.212	60.5%
Yildiz et al. [38]	1.76 (1.43, 2.15)	<0.001	0.046	0.214	63.9%
Yang et al. [10]	1.71 (1.43, 2.05)	<0.001	0.035	0.186	57.3%
Ramezani et al. [29]	1.76 (1.43, 2.17)	<0.001	0.047	0.217	64.0%
Ren et al. [30]	1.69 (1.41, 2.03)	<0.001	0.033	0.182	60.5%
Rojas‐Rodriguez et al. [31]	1.78 (1.42, 2.22)	<0.001	0.054	0.232	62.4%
Sweeting et al. [33]	1.68 (1.41, 2.01)	<0.001	0.030	0.175	58.2%
Xiao et al. [37]	1.86 (1.53, 2.25)	<0.001	0.030	0.174	53.8%
Wells et al. [36]	1.83 (1.46, 2.29)	<0.001	0.056	0.237	64.7%
Lovati et al. [28]	1.61 (1.37, 1.89)	<0.001	0.019	0.136	53.5%

Abbreviations: CI, confidence interval; GDM, gestational diabetes mellitus; OR, odds ratio; PAPP‐A, pregnancy‐associated plasma protein‐A.

### 3.5 Risk of Bias Assessment

The quality ratings of each study using the NOS are shown in Table 1. The scores ranged from 5 to 7, indicating that the studies demonstrated a moderate level of quality. The heterogeneity in study designs, population characteristics, and sampling methodologies underscores the need for careful interpretation of the pooled findings in the meta‐analysis.

### 3.6 Publication Bias Assessment

**FIGURE 3:**
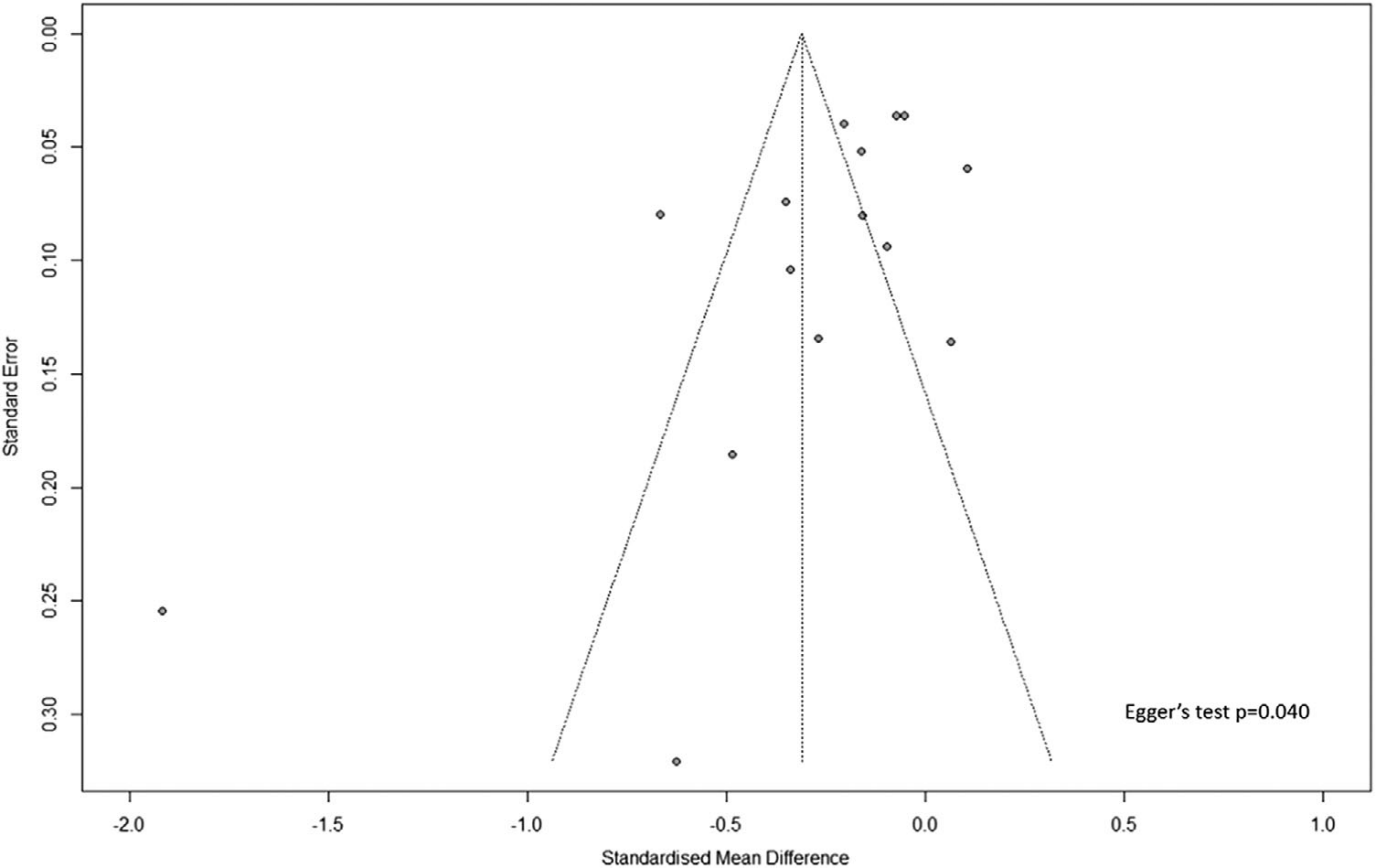
Publication bias assessment for the studies reporting MOM PAPP‐A levels between gestational diabetes mellitus (GDM) and non‐GDM.

Figures 3 and 5 both showed funnel plots of publication bias assessment for the studies on the primary outcomes of the study. Pooled studies regarding the difference in MOM PAPP‐A levels between pregnant women with GDM and non‐GDM had evidence of publication bias according to Egger's regression test (*p* = 0.040) (Figure 3). Similarly, pooled studies for the association between MOM PAPP‐A and GDM risk had evidence of publication bias (Egger's regression test, *p* < 0.001) (Figure 5).

**FIGURE 4:**
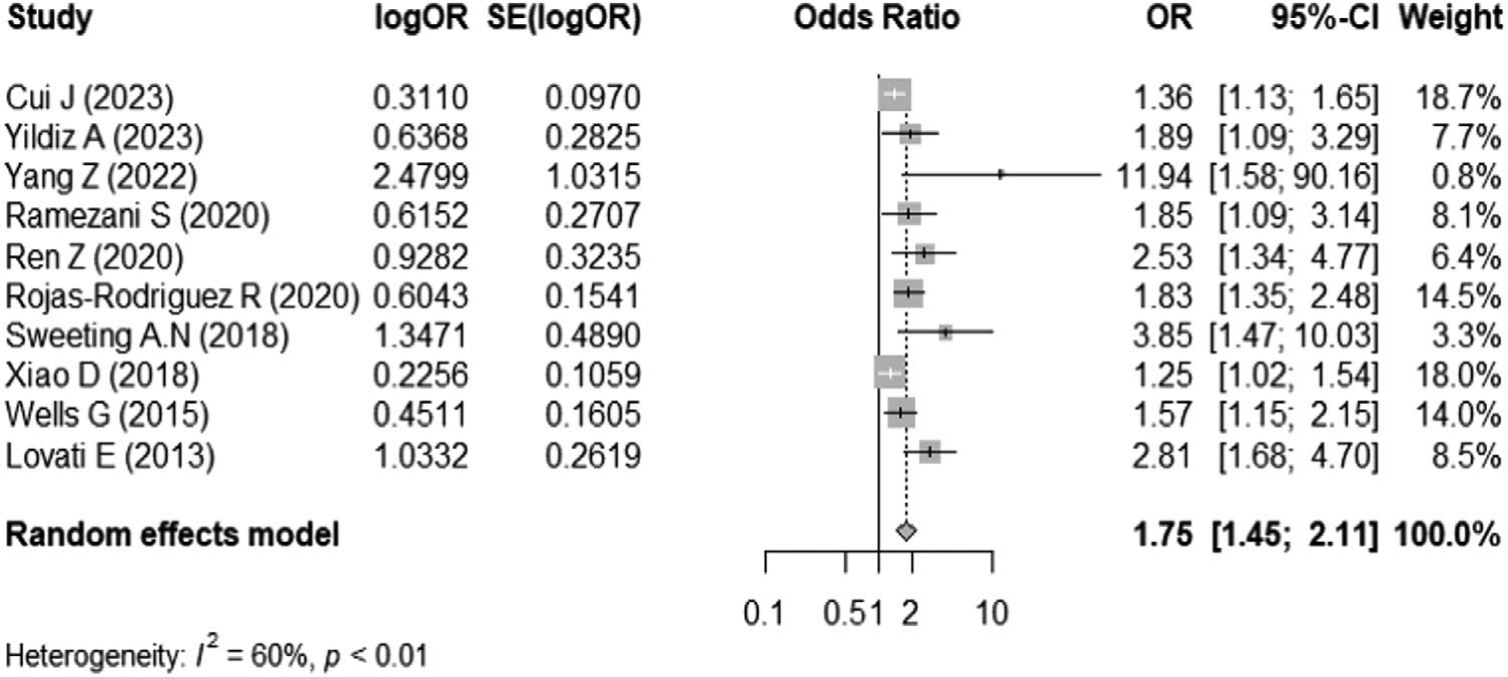
Association between MOM PAPP‐A and gestational diabetes mellitus. CI indicates confidence interval.

**FIGURE 5:**
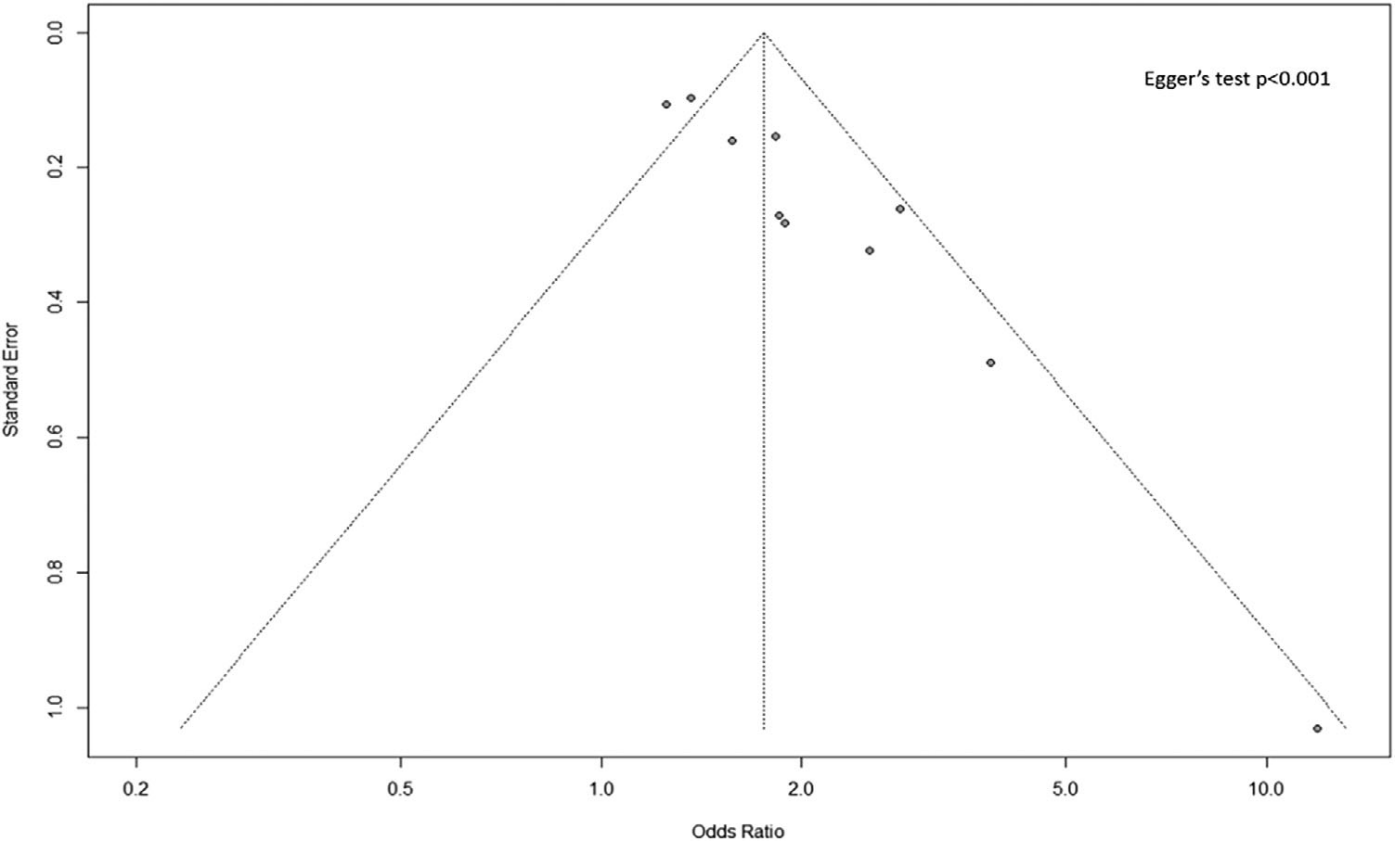
Publication bias assessment for the studies reporting associations between MOM PAPP‐A and gestational diabetes mellitus.

## 4 Discussion

This updated systematic review and meta‐analysis evaluated the predictive utility of PAPP‐A in early pregnancy for the development of GDM. The results revealed that singleton pregnant women with GDM demonstrated significantly lower MOM PAPP‐A levels compared to non‐GDM singleton pregnant women, with a pooled SMD of −0.31. These findings reinforced the inverse association between maternal PAPP‐A levels and GDM risk. Furthermore, women with a low MOM PAPP‐A level in the first trimester had a 1.75‐fold increased risk of developing GDM. These findings underscore the clinical significance of PAPP‐A as a potential predictive biomarker for GDM, warranting further research to validate its utility and explore its integration into early screening strategies.

PAPP‐A, a glycoprotein produced by the placenta during pregnancy, plays a pivotal role in fetal development and is critically involved in the regulation of IGFs for fetal development, as IGFs promote trophoblast invasion and vascularization of the placental bed [11, 12, 13]. When PAPP‐A levels are low, less IGF is available, which can impair early placentation—the process by which the placenta establishes itself in the uterus. This impaired placentation is a key mechanism underlying placental insufficiency, a condition where the placenta fails to adequately supply oxygen and nutrients to the fetus. On the other hand, neonates born to mothers with GDM are predisposed to neonatal hypoglycemia or fetal macrosomia, primarily due to fetal hyperinsulinemia in response to maternal hyperglycemia [40]. Furthermore, GDM is associated with a higher likelihood of cesarean delivery, while infants classified as large for gestational age (LGA) face an elevated risk of shoulder dystocia if delivered vaginally [41, 42].

Recent evidence suggests that fetuses and infants born to women with GDM are at an increased risk of subclinical myocardial dysfunction, which can be early detected using two‐dimensional speckle‐tracking echocardiography, particularly in cases of maternal obesity and poorly controlled diabetes [43, 44, 45]. Maternal diabetes induces intrauterine metabolic stress, impacting fetal myocardial mechanics even before birth. In GDM, abnormal placental function disrupts oxygen and nutrient delivery, contributing to early fetal cardiac remodeling. Chronic exposure to hyperglycemia further exacerbates this process, leading to cardiomyocyte dysfunction, lipotoxicity, and early contractile impairment [44]. Even well‐controlled GDM pregnancies showed subclinical myocardial dysfunction, highlighting the need for early cardiovascular screening in at‐risk fetuses. Increased fetal insulin exposure disrupts normal myocardial contractility, leading to subclinical diastolic dysfunction [43, 45]. These studies provide strong evidence that metabolic control during pregnancy is crucial for preventing long‐term cardiovascular sequelae in offspring.

This updated meta‐analysis expanded previous two meta‐analyses relevant to GDM and PAPP concentrations, both published in 2018 [15, 16]. With only five studies being meta‐analyzed, one of the meta‐analyses was limited by substantial heterogeneity, resulting from diverse adjusted variables, measurements, GDM diagnostic criteria (such as OGTT‐75 g, OGTT‐100 g, and glucose challenge tests [GCT‐50 g]) [16]. Further, high specificity (90%) suggests that if PAPP‐A is low, it strongly indicates GDM risk, but its low sensitivity (55%) means it misses a large proportion of GDM cases. Besides, a threshold effect was detected, meaning the accuracy of PAPP‐A varies depending on the cut‐off values used in different studies. Despite these limitations, the authors concluded that PAPP‐A demonstrated low overall predictive accuracy (area under the curve [AUC]: 0.7) for GDM but suggested its potential utility when integrated with other diagnostic markers or multivariable prediction models [16]. In comparison, this updated meta‐analysis incorporates the latest evidence, substantially expanding the dataset and thereby enhancing the statistical power and robustness of the finding.

As for the other previous meta‐analysis conducted by Donovan et al., examining a total of 83 921 participants, it reported that lower first‐trimester PAPP‐A (MD −0.17; 95% CI −0.24, −0.10) and free β‐hCG (MD −0.04; 95% CI −0.07 ± 0.01) levels were associated with GDM risk. This suggests that placental dysfunction and impaired trophoblast invasion may play a role in early metabolic changes of these biomarkers in GDM. [15]. Lower PAPP‐A levels have also been linked to pre‐eclampsia and fetal growth restriction, conditions associated with placental insufficiency [46]. However, the pooled effect sizes were small. Although statistically significant, the differences in biomarker levels may not be clinically meaningful on their own. Notably, in subgroup analyses, Asian populations showed a weaker association (MD: −0.07) compared to European (MD: −0.21) and Australian (MD: −0.17) populations [15]. Possible ethnic or environmental influences on PAPP‐A levels. Donovan et al. attempted to reduce heterogeneity by stratifying studies based on geographic region, biomarker assay method, and timing of GDM diagnosis, and heterogeneity was reduced after stratifying them. Although we focused specifically on the occurrence rates of GDM, Donovan et al.’s review was conceptually sophisticated, delving into deeper aspects such as outcomes of GDM [15].

### 4.1 Contribution and Clinical Implications

This updated systematic review and meta‐analysis, the largest to date, includes data from over 90 000 pregnancies across multiple regions, incorporating the most recent studies with sensitivity analyses. This comprehensive analysis provides solid evidence on the association between early pregnancy PAPP‐A levels and the risk of developing GDM, further reinforcing the value of incorporating PAPP‐A assessment into practices. However, PAPP‐A should not replace standard GDM diagnostic criteria. Early identification of pregnant individuals at increased risk for GDM allows for targeted monitoring and interventions, potentially reducing the prevalence and severity of GDM‐related complications for both women and children. Further, first‐trimester PAPP‐A screening is routinely conducted for Down syndrome [14], presenting a cost‐effective opportunity for integrating GDM risk stratification into existing prenatal screening protocols, alongside traditional maternal risk factors (e.g., BMI, age, family history). Pregnant women identified as high‐risk based on PAPP‐A could undergo earlier lifestyle interventions (diet, exercise) to mitigate GDM risk [47]. Therefore, PAPP‐A showed its potential value when incorporated into multivariable prediction models or combined with other diagnostic markers.

### 4.2 Limitations and Future Directions

This updated systematic review and meta‐analysis acknowledged limitations. Most studies were case‐control or retrospective in design, possibly introducing selection bias and hardly capturing causal effects. High heterogeneity among studies and detected publication bias present additional concerns, making it difficult to generalize findings. For example, GDM diagnosis misclassification bias and heterogeneity of blood sample collection and management undermined validity. Further, we did not differentiate pregestational dysglycemia, including impaired fasting glucose (IFG) or impaired glucose tolerance (IGT), from GDM. Although some included studies excluded pregestational diabetes, not all explicitly screened for IFG or IGT before pregnancy. This may introduce a risk of misclassification and influence the observed associations. Additionally, there were limited standardized laboratory cut‐off values for PAPP‐A defined among different populations with measurement techniques across studies. The inconsistency in operational definitions remains a limitation that could not be addressed within this meta‐analysis. Although PAPP‐A levels are known to be influenced by various maternal factors, the available data were insufficient to explore these influences through subgroup analysis or meta‐regression, limiting a more comprehensive assessment. Besides, this updated meta‐analysis might exclude non‐English eligible studies and bias the pooled outcomes.

Despite these limitations, the sensitivity analysis indicates robustness in the pooled results, providing confidence in the findings. Future research should focus on minimizing publication bias, standardizing PAPP‐A measurement techniques, and expanding studies to include diverse populations (like advanced maternal age, multiple pregnancy, pregestational dysglycemia, etc) to enhance generalizability. The incorporation of additional clinical studies, especially prospective ones with larger, ethnically diverse prospective cohorts [47], and exploring other relevant metrics like HbA1c, β‐hCG levels, fasting glucose, and insulin resistance markers, will further validate the causative link between PAPP‐A levels and GDM. Furthermore, investigating PAPP‐A's underlying molecular mechanisms could offer insights into novel therapeutic approaches. Collectively, this meta‐analysis reinforces the prognostic value of PAPP‐A levels for GDM, highlighting its potential as a predictive biomarker.

## 5 Conclusion

This updated systematic review and meta‐analysis revealed that a lower PAPP‐A level during the first trimester of pregnancy is associated with a significantly elevated risk of GDM. This finding emphasizes the potential clinical relevance of PAPP‐A as a predictive biomarker, warranting expanded investigation.

## Ethics Statement

The authors confirm that the ethical policies of the journal, as noted on the journal's author guidelines page, have been adhered to. No ethical approval was required as this is a Systematic Review and Meta‐Analysis article with no original research data.

## Conflicts of Interest

The authors declare no conflicts of interest.

## Data Availability

The authors confirm that the data supporting the findings of this systematic review and meta‐analysis are available within the article and its Supporting materials.
